# Revolutionizing RIS Networks: LiDAR-Based Data-Driven Approach to Enhance RIS Beamforming

**DOI:** 10.3390/s25010075

**Published:** 2024-12-26

**Authors:** Ahmad M. Nazar, Mohamed Y. Selim, Daji Qiao

**Affiliations:** Department of Electrical and Computer Engineering, Iowa State University of Science and Technology, Ames, IA 50011, USA; myoussef@iastate.edu (M.Y.S.); daji@iastate.edu (D.Q.)

**Keywords:** LiDAR, beamforming, RIS, GNN, data-driven

## Abstract

Reconfigurable Intelligent Surface (RIS) panels have garnered significant attention with the emergence of next-generation network technologies. This paper proposes a novel data-driven approach that leverages Light Detecting and Ranging (LiDAR) sensors to enhance user localization and beamforming in RIS-assisted networks. Integrating LiDAR sensors into the network will be instrumental, offering high-speed and precise 3D mapping capabilities, even in low light or adverse weather conditions. LiDAR data facilitate user localization, enabling the determination of optimal RIS coefficients. Our approach extends a Graph Neural Network (GNN) by integrating LiDAR-captured user locations as inputs. This extension enables the GNN to effectively learn the mapping from received pilots to optimal beamformers and reflection coefficients to maximize the RIS-assisted sumrate among multiple users. The permutation-equivariant and -invariant properties of the GNN proved advantageous in efficiently handling the LiDAR data. Our simulation results demonstrated significant improvements in sum rates compared with conventional methods. Specifically, including locations improved on excluding locations by up to 25% and outperformed the Linear Minimum Mean Squared Error (LMMSE) channel estimation by up to 85% with varying downlink power and 98% with varying pilot lengths, and showed a remarkable 190% increase with varying downlink power compared with scenarios excluding the RIS.

## 1. Introduction

The advancement of next-generation networks hinges on a thorough analysis of the surrounding environment. By examining static and dynamic events within a specific area, we can fine-tune communication networks to enhance their quality of service (QoS). Effective optimization of these networks necessitates a detailed understanding and preprocessing of environmental characteristics, such as the location and types of obstacles, user locations, and interference levels.

One promising approach to optimizing communication involves preprocessing environmental information to accurately detect users’ locations before transmissions occur. We can improve the power efficiency and minimize interference by directing signals optimally toward these users. A viable method for environmental analysis is the collection of data from sensors mounted on a base station (BS) or at street level. Light Detecting and Ranging (LiDAR) sensors can capture high-resolution visual data at the street level. When mounted on a Reconfigurable Intelligent Surface (RIS)-embedded vehicle, these sensors can gather crucial visual data to optimize network performance.

RISs are advanced metasurfaces composed of numerous small, programmable elements that can manipulate the propagation of electromagnetic waves to enhance communication [1,2,3]. These cost-effective and energy-efficient devices can dynamically adjust the direction, intensity, and phase of reflected data signals by configuring their individual reflecting elements [2,3,4,5]. RIS technology addresses challenges in next-generation networks, including interference mitigation, blockage prediction, and precise localization [6].

LiDAR sensors are particularly advantageous due to their ability to provide highly accurate and precise measurements, even in suboptimal conditions. Unlike camera-based systems, which require extensive processing to determine distances accurately, LiDAR offers direct and precise distance measurements with a lower computational overhead [7,8]. These advantages make LiDAR ideal for capturing reliable environmental data, ensuring the maintenance of high-quality communication networks. LiDAR’s performance degrades less significantly than cameras in adverse weather conditions, such as rain and fog [7,8]. This property makes LiDAR a more reliable choice for applications requiring consistent performance in varying environmental conditions.

LiDAR sensors can detect users within a point cloud using advanced feature extraction techniques, especially when colocated with an RIS. This colocation enables the RIS to be environmentally aware of user locations in multi-path environments [9]. The effectiveness of LiDAR in robust and scale-invariant object detection is exemplified by the Super Fast and Accurate 3D Object Detection (SFA3D) (SFA3D: https://github.com/maudzung/Super-Fast-Accurate-3D-Object-Detection, accessed on 26 August 2024) framework, which employs a Keypoint Feature Pyramid Network (KFPN). This framework underscores LiDAR’s potential in sensing applications, including enhancing user localization for RIS optimization.

The primary contribution of this approach involves utilizing LiDAR-extracted user locations to optimize RIS phase coefficients and BS beamforming vectors. By strategically selecting signal paths corresponding to user positions, we aimed to maximize the achievable user rate while adhering to power constraints. The LiDAR sensor, mounted on an RIS-embedded vehicle, collected 3D visual data for user detection. We then integrated these LiDAR-detected user locations and their pilot sequences into the Graph Neural Network (GNN) model, as introduced in [10].

The GNN maps the received user pilot sequences to optimal BS beamformers and RIS reflection coefficients, maximizing the sum rate among multiple users. The GNN’s permutation-equivariant and -invariant properties ensure that any permutation detected in the user orderings results in a corresponding permutation in the BS beamformers [10]. This characteristic is particularly advantageous when handling LiDAR data, as LiDAR point cloud scans consist of unordered, clustered points. These inputs are processed by the GNN, which maps the detected users and their pilot sequences to optimal beamformers and RIS reflection coefficients.

Despite previous research exploring the use of machine learning and sensor data for RIS optimization, our approach uniquely leverages LiDAR data to achieve high-precision user localization for beamforming and reflection coefficient adjustments. This method addresses existing gaps in the literature by enhancing the accuracy and efficiency of RIS configuration, utilizing novel visual sensor modalities that are crucial for the advancement of next-generation networks.

Our work is organized as follows: Section 2 provides a summary of related work, Section 3 describes the system model and problem formulation, Section 4 and Section 5 present the attained results and discussions, Section 6 discusses future directions, and Section 7 concludes our work.

## 2. Related Work

In recent years, significant advancements have been made in integrating sensing technologies with communication networks, especially LiDAR and RIS, for enhanced network optimization. LiDAR sensors have been extensively studied for their high precision and reliability, enabling real-time object detection and tracking from aerial perspectives when integrated with unmanned aerial vehicles (UAVs). These integrations utilize advanced data fusion techniques and deep learning algorithms, demonstrating LiDAR’s exceptional accuracy in traffic monitoring and network environment optimization [11,12].

RIS technology addresses challenges such as interference, blockage prediction, and precise localization. For instance, a three-stage framework for obtaining channel state information (CSI) with a minimal pilot overhead has shown enhanced robustness in channel estimation through deep learning approaches [6,13]. Moreover, active RIS, capable of amplifying and reflecting signals, surpasses the limitations of passive RIS, including double-fading attenuation, thereby significantly improving the network throughput and coverage for applications such as IoT [14].

Machine learning techniques have been explored extensively to optimize RIS-aided networks further. A deep reinforcement learning algorithm was proposed for beamforming optimization in RIS-aided Multiple-Input–Multiple-Output (MIMO) systems, utilizing location-aware imitation environments to bypass the need for perfect CSI [15]. Additionally, vision-aided RIS systems, which use camera-based visual data to adjust reflection coefficients dynamically, have demonstrated adaptability for beam tracking in dynamic environments [16]. Reviews of optimization methods and AI-based techniques emphasize balancing the solution quality and computational complexity to optimize phase shifts and other network resources effectively [17].

RIS technology has also been investigated for vehicular networks, where it enhances communication by reflecting, refracting, and focusing signals programmatically to significantly improve the quality and efficiency in dynamic environments [18]. Integrating RIS with LiDAR data further improves user localization and beamforming precision. By leveraging high-resolution spatial data from LiDAR sensors, RIS phase shifts and base station beamforming vectors can be optimized to achieve substantial performance gains.

The relevance of integrating LiDAR with GNNs for optimizing RIS configurations is underscored by the ability of GNNs to process complex environmental data and adapt dynamically to real-time changes. These capabilities make GNNs a robust solution for next-generation wireless communication systems, providing an innovative approach to tackling the challenges of high user density and dynamic network environments.

## 3. Materials and Methods

**Figure 1:**
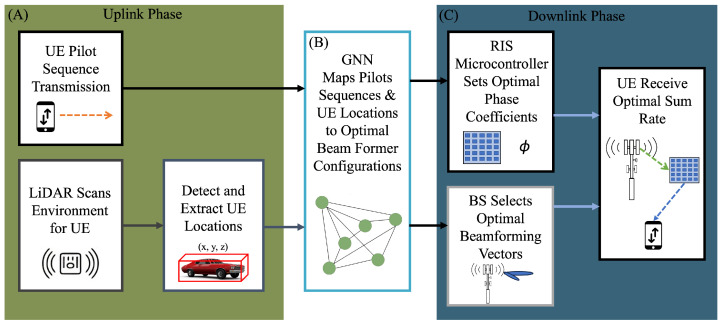
The general flow of the proposed system where (**A**) shows the UE pilot transmissions sent to the GNN in the uplink phase, and LiDAR captures the environment for user detection. In (**B**), the GNN optimizes the RIS phase shifts and BS beamforming vectors. In (**C**), the RIS microcontroller is reconfigured for the optimal phase coefficients, and the BS selects the optimal beamformers for the optimal UE downlink sumrate.

This section introduces the proposed approach’s system model, which integrates a GNN with LiDAR data. The high-level system, as shown in Figure 1, shows the general flow of the proposed approach. In Figure 1A, UE pilot transmissions are sent to the GNN in the uplink phase, and LiDAR captures the environment for user detection. In Figure 1B, the GNN learns the mapping of the user locations and pilot sequences to output optimized RIS phase shifts and BS beamforming vectors. Finally, in Figure 1C, the RIS microcontroller adjusts for optimal phase coefficients, and the BS selects optimal beamformers to optimize the maximum–minimum achievable UE downlink sumrate. The GNN utilizes uplink pilot signals and the spatial coordinates of detected users to maximize the minimum achievable user sum rate. The sumrate is achieved by optimizing the RIS phase coefficients and beamforming vectors.

### 3.1. Scenario

**Figure 2:**
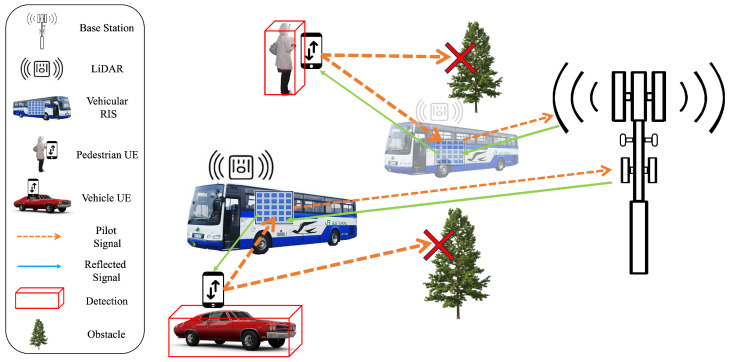
Dynamic environment scenario with blockages, active users, and detection framework uplink pilot transmissions for optimized downlink sumrate. UE attempt to send uplink pilot sequences, as indicated by the orange arrow, directly to the BS but are blocked by an obstacle; the UE then use the RIS in LoS with the BS and the UE to reflect the uplink pilot sequence. The BS uses the received uplink pilot sequence to optimal beamforming vectors and RIS phase coefficients for optimal UE sumrate in the downlink as shown by the green arrow.

The scenario considered a Multiple-Input–Single-Output (MISO) downlink system with *U* single-antenna static users in Line of Sight (LoS) with an *N*-element passive RIS mounted on a vehicle, as shown in Figure 2. An *M*-antenna BS is considered for the approach serving *U* users and is in LoS with the RIS. Given the dynamic nature of the RIS and LiDAR-mounted vehicle and the high frequency of point cloud scans, user positions are continuously updated with each scan. While our approach considers static users, not mobile users, the frequency of scans and detections ensures accurate tracking even for mobile users.

Mounting the RIS on a vehicle enables dynamic positioning and the LiDAR colocated with the RIS scans the environment and provides real-time user locations with respect to both the RIS and the BS. The RIS continuously updates the phase coefficients to accurately transmit signals from the BS to the UE. The communication channel operates under block-fading conditions and is affected by multi-user interference, which is treated as additional noise. The thermal noise generated by RIS components, including phase shifters and controllers, is minimal due to their low power consumption and does not actively amplify the reflected signals. While RIS thermal noise could theoretically act as an additional noise source scaled by the channel gain, this contribution is assumed negligible relative to the dominant additive receiver noise in our model. This assumption aligns with established RIS modeling practices in the literature, where environmental noise and receiver noise are the primary considerations. In this setup, the BS remains stationary, while the vehicle equipped with the RIS is assumed to be positioned at the origin.

Data transmission occurs during both the uplink and downlink phases. In the uplink phase, users send pilot sequences to the BS. A vehicle with an RIS scans the environment using LiDAR to detect users with the SFA3D object detection framework. The Cartesian coordinates of the detected users, represented as (x,y,z), along with their pilot sequences from the uplink phase, are transmitted to the BS. These inputs serve as parameters for a GNN, which maps the user pilot sequences and their detected locations to optimal beamformers and phase coefficients to maximize the sum rate across *U* users.

The GNN possesses permutation-equivariant and -invariant properties; this means that rearranging the order of users results in a corresponding rearrangement of the BS beamformers. Additionally, this property ensures that the reflective pattern matrices of the RIS remain fixed in relation to these input permutations. These characteristics are particularly beneficial when dealing with LiDAR data, as LiDAR point cloud scans consist of an unordered, clustered sets of points.

### 3.2. Beamforming and RIS Phase Coefficient Scheme

The uplink transmission phase estimates the channel, and the approach assumes channel reciprocity [10,19]. The BS antennas are arranged in a uniform linear array aligned with the *x*-axis. The RIS comprises a uniform rectangular array configured as n×n and is positioned on the (y,z)-plane. For baseline purposes, the RIS was configured in a uniform 10×10 rectangular array arrangement with N=100 elements. The uplink channel vectors from user *u* to the BS are denoted as $h_{u}^{d}\in C^{M}$ such that

(1) $$h_{u}^{d}=\beta_{0,u}{\tilde{h}}_{u}^{d},$$

where ${\tilde{h}}_{u}^{d}∼CN\left(0,I\right)$, the pathloss in dB between a user and the BS was $\beta_{0,u}=32.6+36.7\log\left(d_{u}^{\mathrm{BS}-U}\right)$, and $d_{u}^{\mathrm{BS}}$ is the distance between user *u* and the BS [10].

Based on the scenario definition, the RIS is deployed at a location in LoS with the users and the BS; therefore, the channel between the RIS and user *u* and between the BS and the RIS are modeled as Rician fading channels. The Rician channel model is commonly used for LoS scenarios in wireless communication systems and has been validated in prior research for RIS-based setups [20]. The uplink channel vectors from user *u* to the RIS are recognized as $h_{u}^{r}\in C^{N}$ such that

(2) $$h_{u}^{r}=\beta_{1,u}\left(\sqrt{\frac{\varepsilon }{1+\varepsilon }}{\tilde{h}}_{u}^{r,\mathrm{LOS}}+\sqrt{\frac{1}{1+\varepsilon }}{\tilde{h}}_{u}^{r,\mathrm{NLOS}}\right),$$

where the pathloss between a user *u* and the RIS was $\beta_{1,u}=30+22\log\left(d_{u}^{\mathrm{RIS}-U}\right)$ and $d_{u}^{\mathrm{RIS}}$ is the distance between the user and the RIS [10]. This equation represents the uplink channel vectors from the user to the RIS, incorporating both LoS and Non-Line of Sight (NLoS) components, weighted by a Rician factor to account for the mixed propagation environment [10].

The *n*-th element steering vector of the RIS is denoted as

(3) $${\left[a_{\mathrm{RIS}}\left(\Phi_{3,u}^{*},\theta_{3,u}^{*}\right)\right]}_{n}=e^{j\frac{2\pi d^{\mathrm{RIS}}}{\lambda_{c}}\left\{i_{1}\left(n\right)\sin\left(\Phi_{3,u}^{*}\right)\cos\left(\theta_{3,u}^{*}\right)+i_{2}\left(n\right)\sin\left(\theta_{3,u}^{*}\right)\right\}},$$

where $\Phi_{3,u}^{*}$ and $\theta_{3,u}^{*}$ are the azimuth and elevation angles of arrival from the user *u* to the RIS, $\lambda_{c}$ is the carrier wavelength, $d^{\mathrm{RIS}}$ is the distance between two adjacent elements, $i_{1}\left(n\right)=\mathrm{mod}\left(n-1,10\right)$, and $i_{2}\left(n\right)=\left\lfloor n-1/10\right\rfloor$ [10]. For the simulations, $\frac{2\pi d^{\mathrm{RIS}}}{\lambda_{c}}$ was assumed to be 1. The LoS component of $h_{u}^{r}$ is a function of RIS and *U* users’ locations in LoS with each other such that

(4) $${\tilde{h}}_{u}^{r,\mathrm{LOS}}=a_{\mathrm{RIS}}\left(\Phi_{3,u}^{*},\theta_{3,u}^{*}\right),$$

and user *u*’s location is $\left(x_{u},y_{u},z_{u}\right)$, as given by the detection framework in Section 3.3. The location of the RIS is $\left(x_{\mathrm{RIS}},y_{\mathrm{RIS}},z_{\mathrm{RIS}}\right)$, and the distance from user *u* to the RIS is $d_{u}^{\mathrm{RIS}-U}$ [10]. The RIS-mounted vehicle continuously updates the user locations using LiDAR, which are sent dynamically to the GNN for real-time beamforming optimization. These locations are then expressed through the following [10]:

(5) $$\begin{array}{cc} \sin\left(\Phi_{3,u}^{*}\right)\cos\left(\theta_{3,u}^{*}\right)= & \frac{y_{u}-y^{\mathrm{RIS}}}{d_{u}^{\mathrm{RIS}-U}}, \end{array}$$

(6) $$\begin{array}{cc} \sin(\theta_{3,u}^{*})= & \frac{z_{u}-z^{\mathrm{RIS}}}{d_{u}^{\mathrm{RIS}-U}}. \end{array}$$

The uplink channel matrix from the RIS to the BS is recognized as $G\in C^{M\times N}$ [10]. G is composed of LoS and NLoS components ${\tilde{G}}^{\mathrm{LOS}}$ and ${\tilde{G}}^{\mathrm{NLOS}}$, respectively; a Rician factor ε, and a pathloss $\beta_{2}=30+22\log\left(d_{u}^{\mathrm{RIS}-\mathrm{BS}}\right)$ between RIS and BS in dB such that

(7) $$G=\beta_{2}\left(\sqrt{\frac{\varepsilon }{1+\varepsilon }}{\tilde{G}}^{\mathrm{LOS}}+\sqrt{\frac{1}{1+\varepsilon }}{\tilde{G}}^{\mathrm{NLOS}}\right).$$

The steering vector of the BS is expressed as the following:

(8) $$a_{\mathrm{BS}}\left(\Phi_{1}^{*},\theta_{1}^{*}\right)=\left[1,\ldots ,e^{j\frac{2\pi \left(M-1\right)d^{\mathrm{BS}}}{\lambda_{c}}\cos\left(\Phi_{1}^{*}\right)\cos\left(\theta_{1}^{*}\right)}\right],$$

where $\Phi_{1}^{*}$ and $\theta_{1}^{*}$ are the azimuth and elevation angles of arrival to the BS, $d^{\mathrm{BS}}$ is the distance between two adjacent antennas, and $\frac{2\pi d^{\mathrm{BS}}}{\lambda_{c}}$ was assumed to be 1 [10]. The LoS component of the BS-RIS channel can then be expressed as

(9) $${\tilde{G}}^{\mathrm{LOS}}=a_{\mathrm{BS}}\left(\Phi_{1}^{*},\theta_{1}^{*}\right)a_{\mathrm{RIS}}{\left(\Phi_{2}^{*},\theta_{2}^{*}\right)}^{H},$$

where $\Phi_{2}^{*}$ and $\theta_{2}^{*}$ are the azimuth and elevation angles of departure from the RIS to the BS [10]. The location of the BS at $\left(x_{\mathrm{BS}},y_{\mathrm{BS}},z_{\mathrm{BS}}\right)$ can then be used to obtain the following:

(10) $$\begin{array}{cc} \cos\left(\Phi_{1}^{*}\right)\cos\left(\theta_{1}^{*}\right)= & \frac{x^{\mathrm{RIS}}-x^{\mathrm{BS}}}{d^{\mathrm{BS}-\mathrm{RIS}}}, \end{array}$$

(11) $$\begin{array}{cc} \sin\left(\Phi_{2}^{*}\right)\cos\left(\theta_{2}^{*}\right)= & \frac{y^{\mathrm{BS}}-y^{\mathrm{RIS}}}{d^{\mathrm{BS}-\mathrm{RIS}}}, \end{array}$$

(12) $$\begin{array}{cc} \sin(\theta_{2}^{*})= & \frac{z^{\mathrm{BS}}-z^{\mathrm{RIS}}}{d^{\mathrm{BS}-\mathrm{RIS}}}. \end{array}$$

**Figure 3:**
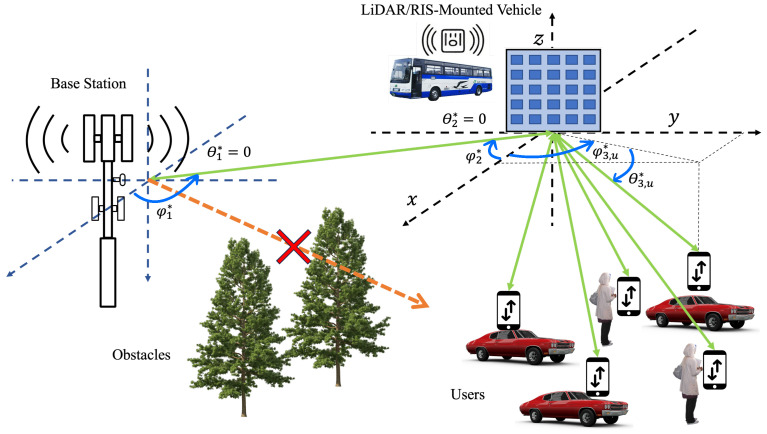
Definition of angles in relation to the RIS plane, user positions, and beamforming vectors. The RIS comprises a uniform rectangular array configured as n×n and is positioned on the (y,z)-plane. The direct BS-to-UE signal is blocked by an obstacle as shown by the orange arrow, and the UE-RIS-BS communication is established to optimize the UE sumrate as indicated by the green arrow.

The system model, depicted in Figure 3, clearly defines the angles, visually representing their relationship to the RIS plane, user positions, and beamforming vectors.

The NLoS components ${\tilde{h}}_{u}^{r,\mathrm{NLOS}}$ and ${\tilde{G}}^{\mathrm{NLOS}}$ are modeled as independent and identically distributed (i.i.d) Gaussian distributions. In the downlink direction, the channel matrices are assumed to be the transposes of the uplink channels, based on the principle of channel reciprocity [10,19].

The BS’s beamforming scheme employs a beamforming vector $w_{u}\in C^{M}$ to transmit the data symbol $s_{u}\in C$ to user *u*. The optimization problem requires that the beamforming vector $w_{u}$ complies with the power constraint $P_{d}$, where $\sum_{u=1}^{U}{∥w_{u}∥}_{2}^{2}\leq P_{d}$ [10].

The RIS reflection coefficients are denoted as $\phi =[\phi_{1},\ldots ,\phi_{N}]$, where $\phi_{m}$ denotes the reflection coefficient of the *m*-th reflecting element. The received signal $r_{u}$ for user *u* is expressed as

(13) $$r_{u}=\sum_{j=1}^{U}{\left(h_{u}^{d}+A_{u}\phi \right)}^{⊤}w_{j}s_{j}+n_{u},$$

where the cascaded channel from user *u* to the RIS and then to the BS is represented by $A_{u}=G\mathrm{diag}\left(h_{u}^{r}\right)$ [10]. The term $n_{u}∼CN\left(0,\sigma_{0}^{2}\right)$ denotes the multi-user interference modeled as additive white Gaussian noise. User *u*’s achievable rate $R_{u}$ is given by

(14) $$R_{u}=\log\left(1+\frac{{\left|{\left(h_{u}^{d}+A_{i}\phi \right)}^{⊤}w_{u}\right|}^{2}}{\sum_{i=1,i\neq u}^{U}{\left|{\left(h_{k}^{d}+A_{u}\phi \right)}^{⊤}w_{i}\right|}^{2}+\sigma_{0}^{2}}\right),$$

where the channel follows a block-fading model such that the channel coefficients change dynamically and independently from block to block and are static during a coherence block [10].

The maximum network utility $U(R_{1},\ldots ,R_{U})$ is a function of the achievable rates for *U* users. This utility can be jointly optimized by adjusting the beamforming vectors $w_{u}$ of the BS and the phase shifts ϕ of the RIS. In this optimization process, the network utility maximizes the minimum rate, expressed as $\min_{u}R_{u}$, and the total sum rate $\sum_{u=1}^{U}R_{u}$.

Channel estimation occurs during a pilot transmission phase in the uplink, leveraging channel reciprocity [10,19]. It requires prior knowledge of $A_{u}$ and the channel vector $h_{u}^{d}$ for optimization of the transmit beamformers at the BS and RIS reflection coefficients. The uplink pilot transmission phase is utilized to acquire information about the cascaded channel matrix $A_{u}$ and the channel vector $h_{u}^{d}$. The pilot sequences are represented as $x_{u}\left(\ell \right)$, where ℓ=1,…,L indicates the time slots of the pilot phase. These sequences are transmitted to the BS via the RIS, resulting in a received signal y(ℓ), which is expressed as

(15) $$\begin{array}{cc} y(\ell ) & =\sum_{u=1}^{U}\left(h_{u}^{d}+G\mathrm{diag}\left(\phi \left(\ell \right)\right)h_{u}^{r}\right)x_{u}\left(\ell \right)+n\left(\ell \right),\\ \; & =\sum_{u=1}^{U}\left(h_{u}^{d}+A_{u}\phi \left(\ell \right)\right)x_{u}\left(\ell \right)+n\left(\ell \right), \end{array}$$

where diag(ϕ(ℓ)) is a diagonal matrix containing the RIS phase shifts ϕ(ℓ) on its diagonal at time slot *ℓ* of the pilot transmission phase [10]. The additive white Gaussian noise, which represents multi-user interference, is denoted as $n\left(\ell \right)∼CN\left(0,\sigma_{1}^{2}I\right)$.

The optimal beamforming vector $w_{u}$ bypasses the channel estimation by linking the pilot transmission phase to the optimized transmission strategy to maximize the utility. Specifically, $w_{u}$ is determined by solving the following optimization problem:

(16) $$\begin{array}{cc} \underset{\left(W,\phi )=g(Y\right)}{\mathrm{maximize}} & E\left[U\left(R_{1}\left(\phi ,W\right),\ldots ,R_{U}\left(\phi ,W\right)\right)\right],\\ \;\mathrm{subject}\;\mathrm{to}\; & \sum_{u}{∥w_{u}∥}^{2}\leq P_{d}\\ \; & \left|\phi_{i}\right|=1,i=1,2,\cdots ,N \end{array}$$

where $Y=\left[y\left(1\right),y\left(2\right),\ldots ,y\left(L\right)\right]\in C^{M\times L}$ represents the received pilots, while the beamforming matrix at the BS is denoted by $W=[w_{1},\ldots ,w_{u}]$ [10]. The received pilots are mapped during the uplink transmission phase to both W and the phase shift vector ϕ by g(·). The network utility function is U(·). The GNN aims to parameterize g(·) and learn the neural network parameters from the received data.

In this context, pilot sequences are transmitted for channel measurement. The pilot transmission phase divides the total training time slots *L* into τ subframes, each of which containing $L_{0}=U$ such that $L=\tau L_{0}$. Users send length $L_{0}$ pilot sequences to the BS every τ subframes, denoted as $x_{u}^{H}=\left[x_{u}\left(1\right),x_{u}\left(2\right),\ldots ,x_{u}\left(L_{0}\right)\right]$. These sequences are orthogonal to one another, facilitating easier decorrelation at the BS. The RIS phase shifts remain static within the subframes but are dynamic across different subframes [10].

The received pilots in frame *t* are decorrelated at the BS in each subframe to match each user’s pilot [10]. These received pilots are denoted as

(17) $$\bar{Y}\left(t\right)=[y\left(\left(t-1\right)L_{0}+1,\ldots ,y\left(tL_{0}\right)\right],$$

which can then be further expressed as

(18) $$\bar{Y}\left(t\right)=\sum_{u=1}^{U}\left(h_{u}^{d}+A_{u}\bar{\phi }\left(t\right)\right)x_{u}^{H}+\bar{N}\left(t\right),\;t=1,\cdots ,\tau$$

and $\bar{N}\left(t\right)$ has columns that are independently distributed such that $\bar{N}\left(t\right)∼\mathrm{CN}\left(0,\sigma_{1}^{2}I\right)$ [10]. The user’s uplink transmission ${\bar{y}}_{u}\left(t\right)$ then consists of the following:

(19) $$\begin{array}{cc} {\bar{y}}_{u}\left(t\right)= & \frac{1}{L_{0}}\bar{Y}\left(t\right)x_{u}=h_{u}^{d}+A_{u}\bar{\phi }\left(t\right)+\bar{n}\left(t\right), \end{array}$$

(20) $$\begin{array}{cc} ≜ & F_{k}q\left(t\right)+\bar{n}\left(t\right), \end{array}$$

where $\bar{n}\left(t\right)=\frac{1}{L_{0}}\bar{N}\left(t\right)x_{u}$, the combined channel matrix is $F_{k}≜\left[h_{u}^{d},A_{u}\right],$ and the combined phase shifts are $q\left(t\right)≜{\left[1,\bar{\phi }{\left(t\right)}^{⊤}\right]}^{⊤}$ [10]. The received pilots across all frames are represented by ${\tilde{Y}}_{u}=\left[{\bar{y}}_{u}\left(1\right),\ldots ,y_{u}\left(\tau \right)\right]$ and expressed as

(21) $${\tilde{Y}}_{u}=F_{u}Q+\tilde{N},$$

where $Q=[\bar{q}\left(1\right),\ldots ,q\left(\tau \right)]$ and $\tilde{N}=\left[\bar{n}\left(1\right),\ldots ,\bar{n}\left(\tau \right)\right]$. $F_{u}$ is then estimated by the minimum mean-squared error (MMSE) estimator using

(22) $$\underset{f(\cdot )}{\mathrm{minimize}}\;E\left[∥f\left({\tilde{Y}}_{u}\right)-F_{u}{∥}_{F}^{2}\right],$$

which has an optimal solution provided by the following computationally intensive equation:

(23) $$f\left({\tilde{Y}}_{u}\right)=E\left[F_{u}|{\tilde{Y}}_{u}\right].$$

The Linear MMSE (LMMSE) is a conventional baseline for comparing user sumrates against the GNN [10]. The LMMSE linearly constrains the estimator f(·), resulting in the following:

(24) $$\begin{array}{cc} \hat{F_{u}} & =\left({\tilde{Y}}_{u}-E\left[{\tilde{Y}}_{u}\right]\right){\left(E[{\left({\tilde{Y}}_{u}-E\left[{\tilde{Y}}_{u}\right]\right)}^{H}\left({\tilde{Y}}_{u}-E\left[{\tilde{Y}}_{u}\right]\right)]\right)}^{-1}\\ \; & \;E\left[{\left({\tilde{Y}}_{u}-E\left[{\tilde{Y}}_{u}\right]\right)}^{H}\left(F_{u}-E\left[F_{u}\right]\right)\right]+E\left[F_{u}\right], \end{array}$$

where ${\hat{F}}_{u}$ estimates $A_{u}$ and $h_{u}^{d}$ only when the rows of $F_{u}$ and ${\tilde{N}}_{u}$ are i.i.d Gaussian distributed.

The GNN acquires the graph representation vector by traversing several layers: an initialization layer, multiple aggregation and combination layers (indexed by *D*), a linear layer, and a final normalization layer. The process begins with input features from the user node forming the initial representation vector. Aggregation and combination rules transform this vector through the *D* layers. A linear layer then refines and normalizes the representation to map the beamformer matrix *W* and the phase shifts ϕ.

The GNN uses user pilots $\bar{Y}\left(t\right)$ concatenated with detected user locations (*x*-, *y*-, *z*-coordinates) as inputs. These inputs pass through a fully connected neural network layer with an element-wise mean function. The resulting vectors are processed through *D* layers, which update the GNN to produce optimal RIS phase coefficients ϕ and beamformers $w_{u}$. The *D* layers aggregate and combine outputs from neighboring nodes using permutation-invariant functions, differing for the RIS and the set of users *U*.

**Figure 4:**
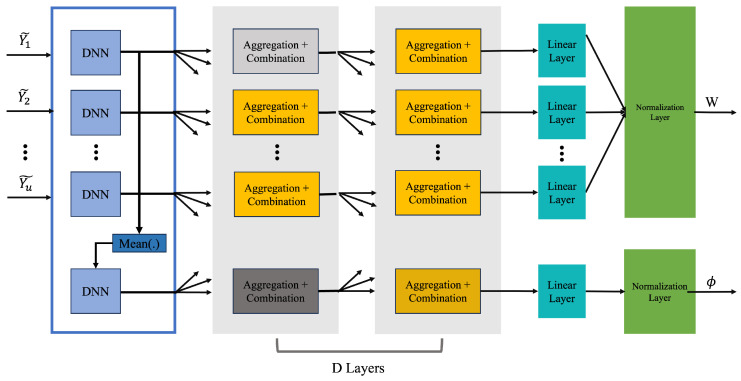
High-level GNN architecture mapping user pilots to beamforming vectors and phase shifts. Inputs pass through a fully connected neural network, *D* combination and aggregation layers, a linear layer, and a normalization layer to output phase coefficients and optimal beamforming vectors.

The output of the *D* layers is passed to a linear layer and then to a normalization layer, yielding the reflection coefficients and the optimal beamformer matrix. The real and imaginary components of the achievable rates are separated to train the GNN, adjusting weights to maximize the network utility. The optimal beamformers and phase coefficients are *W* and ϕ, respectively. Detailed GNN architecture explanations and training processes are provided in [10], and an overview of the neural network architecture is shown in Figure 4.

The second phase utilizes information transmitted to the base station. The GNN optimizes RIS phase shifts to reflect signals toward user *u* accurately. The optimization criteria involve selecting the best beam vector while satisfying a power requirement *P*.

### 3.3. Super Fast and Accurate 3D Object Detection Based on 3D LiDAR Point Clouds (SFA3D)

The “KITTI Vision Benchmark Suite for 3D Object Detection Evaluation in 2017”, referred to as the KITTI Dataset, is a vital resource for assessing detection algorithms [21]. This dataset combines LiDAR, radar, and stereo camera data collected from a driving platform, focusing on three primary object classes: cars, pedestrians, and cyclists. It is annotated with ground truth labels, which include accurate 3D bounding boxes for the objects, alongside high-resolution images and 3D point clouds. The dataset covers a variety of scenarios, presenting challenging variations in scenes, lighting conditions, weather, and object poses. The KITTI Dataset is the primary reference for analyzing detected active users in the environment [21].

The 3D object detection framework SFA3D utilizes a ResNet-based KFPN designed for robust performance and object detection in LiDAR-based environments [22,23]. The KFPN processes Bird’s-Eye View (BEV) image maps as its input, representing 3D LiDAR point clouds with encoded height, intensity, and density information. KFPN extends Feature Pyramid Networks (FPNs) by upsampling feature map outputs to match the largest feature map output within the set of outputs, computing a weighted average of that feature map, and outputting a single feature map with the highest confidence values for the detected objects.

The BEV input $I\in R^{W\times H\times 3}$ represents point clouds from the KITTI Dataset as an RGB image. This image is downsampled with a factor S=4 and processed through the KFPN’s convolutional layers to extract feature maps $f_{i}$ of decreasing size. These feature maps are upscaled and combined through a weighted average, resulting in a single feature map *F* with the highest confidence values for detected objects. *F* is represented as

(25) $$F=\mathrm{weighted}\_\mathrm{average}(\mathrm{upscale}\_\mathrm{to}(\max(f_{i})),$$

The output of SFA3D, $F_{\mathrm{out}}$, consists of the prediction of multi-dimensional outputs, including heatmaps for main centers, center offsets, heading angles (yaws), object dimensions (length, width, height), and z-coordinates. The output-detected object representation estimates seven degrees of freedom parameters: 〈cx,cy,cz,l,w,h,θ〉. These parameters define the object’s spatial characteristics, encompassing the bounding box center coordinates, bounding box dimensions, and heading angle. KFPN outputs multi-dimensional information crucial for precise object detection, all of which is processed in a multi-layer CNN.

**Figure 5:**
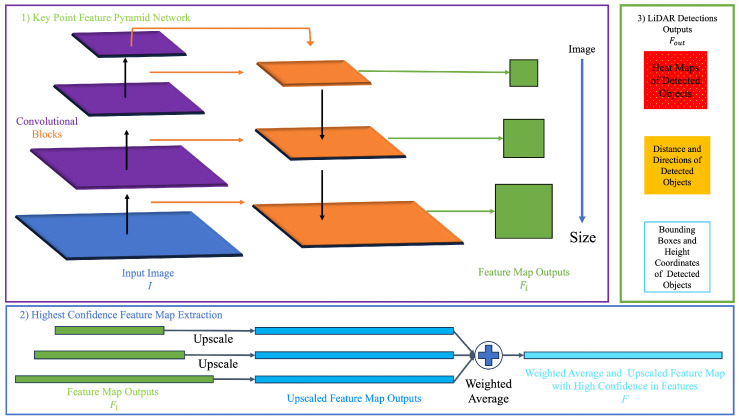
Overview of SFA3D framework model and components.

The KFPN is scale-invariant for point cloud object detection, making it particularly effective for handling multiple LiDAR devices, merged viewpoints, and distant objects. This scale-invariant property means that object feature extraction and detection can be performed regardless of the object’s size. This advantage is beneficial when dealing with data from multiple LiDAR devices positioned at different viewpoints and detecting objects far from the LiDAR’s origin. Figure 5 summarizes and provides an overview of the SFA3D network architecture.

The technical goal is to associate the locations of vehicular or pedestrian users detected by the SFA3D framework using the KITTI Vision Benchmark Dataset with users served by a vehicular RIS in LoS conditions with the BS. This goal involves leveraging the detection capabilities of the chosen model to recognize users within the dataset accurately. The model outputs coordinates converted into real-world (x,y,z)-coordinates, allowing us to map the detected users in the KITTI Dataset to their spatial locations relative to the BS and the RIS. Each detected object corresponds to a specific user, denoted as user *u*, whose location is given by $\left(x_{u},y_{u},z_{u}\right)$. These locations are subsequently incorporated as supplementary input into the GNN. This work assumes LoS conditions for RIS-to-user communication. However, in NLoS scenarios between the user and the RIS, LiDAR can accurately determine user locations using its point-cloud-processing capabilities. Even when the direct communication path is blocked, whether between the user and the RIS or the BS, LiDAR can detect the user’s spatial coordinates. This information allows the system to calculate an optimal indirect reflection path via the RIS to redirect the signal toward the user efficiently. Additionally, the LiDAR can be colocated with either the BS or the RIS. To improve the detection, the LiDAR can also be positioned at street level to capture users in NLoS conditions better. Another approach to improve the coverage for NLoS users is to deploy multiple LiDARs within the BS coverage area, ensuring a more comprehensive and accurate detection of user locations.

This approach highlights the effectiveness of LiDAR technology in achieving high precision and robustness in object detection, which aligns with our method of using LiDAR data for accurate user localization in RIS-assisted communication networks. By integrating the locations detected by LiDAR into the GNN, the system can utilize precise environmental data, enabling optimized beamforming and adjustments to the reflection coefficients, ultimately enhancing the overall sum rate.

## 4. Results

We evaluated the proposed LiDAR-aided GNN framework using four key experiments: we (1) analyzed the impact of varying the downlink power on the achievable sumrate, (2) examined the effect of the uplink power, (3) studied the effect of varying the pilot sequence lengths, and (4) explored the impact of varying the number of RIS elements. All experiments were conducted under realistic conditions, with RIS elements and user locations derived from a vehicular-mounted setup. The results compare the performance of the proposed method to a baseline LMMSE estimator and a location-exclusive GNN to highlight its advantages.

The simulation parameters, as listed in Table 1, were chosen to emulate a realistic environment. The users’ locations are extracted using SFA3D and stored as 〈x,y,z〉-tuples, representing the bounding box center locations for the vehicular-mounted RIS. Scenarios were extracted based on 1000 samples containing five users from the KITTI Dataset.

### 4.1. Experiment 1: Varying Downlink Transmission Power

The first experiment examined how the downlink power levels influenced the achievable sumrate in the proposed RIS-assisted network. The uplink power level was fixed at $P_{\mathrm{uplink}}=5$ dBm, while the downlink power varied across the set $\left\{P_{\mathrm{downlink}}\right\}=\left\{0,2.5,5,7.5,10,12.5,15,17.5,20\right\}$ dBm. The user pilot sequence length was set to L=15. The number of RIS elements was set to N=100. LiDAR-detected user locations were integrated into the GNN framework and compared with a location-exclusive GNN and an LMMSE channel estimator.

**Figure 6:**
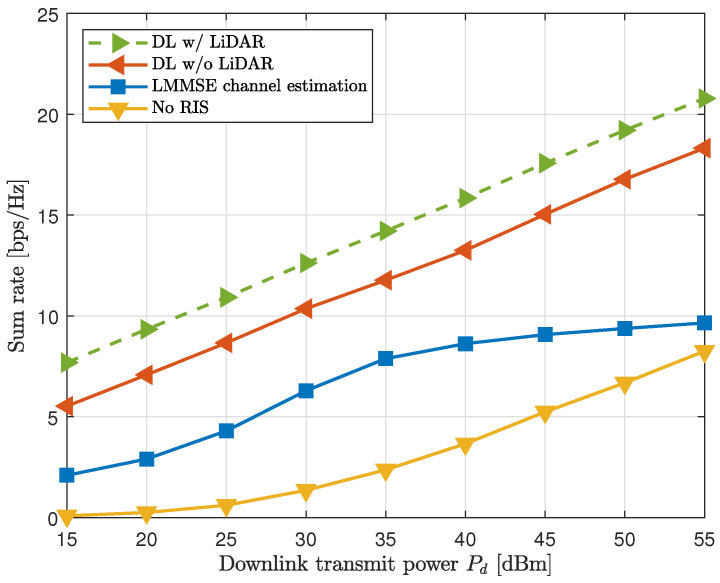
Sumrate comparison with various downlink powers $P_{\mathrm{downlink}}$.

As shown in Figure 6, the GNN with LiDAR-detected locations consistently outperformed the other methods. Specifically, the location-inclusive GNN achieved a 16% higher sumrate than the location-exclusive GNN and an 85% improvement over the LMMSE estimator. The most significant improvement, 190%, was observed when comparing the LiDAR-aided GNN with the scenario that excluded the RIS entirely. These results emphasize the importance of leveraging high-resolution spatial information from LiDAR to optimize beamforming and RIS phase coefficients.

### 4.2. Experiment 2: Varying Uplink Transmission Power

The second experiment evaluated the influence of the uplink power on the achievable sumrate, which is critical for understanding how uplink pilots impact GNN-based optimization. The downlink power was set to $P_{\mathrm{downlink}}=20$ dBm, and the pilot sequence length was fixed at L=15. The uplink power was varied across the set $\left\{P_{\mathrm{uplink}}\right\}=\left\{0,2.5,5,7.5,10,12.5,15,17.5,20\right\}$ dBm. The number of RIS elements was set to N=100. The evaluation was conducted across multiple configurations, including LiDAR-detected locations, excluding them, using the LMMSE estimator, and excluding the RIS entirely.

**Figure 7:**
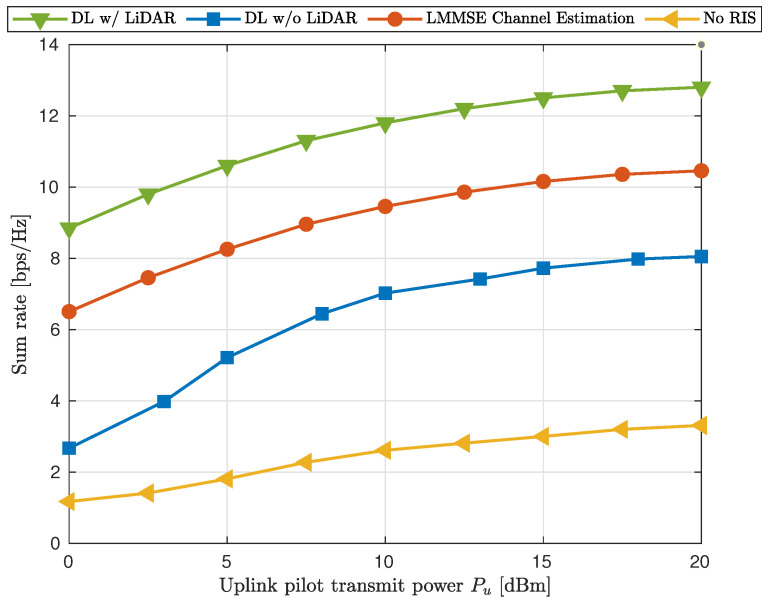
Sumrate comparison with various uplink powers $\left\{P_{\mathrm{uplink}}\right\}$.

The GNN with LiDAR-detected locations consistently achieved higher sumrates with varying uplink transmission powers, as shown in Figure 7. The performance gap between including and excluding the LiDAR data was 25.4%, and between the LiDAR-aided GNN and the LMMSE, the improvement was 73.1%. The improvement when using an RIS-assisted LiDAR-aided GNN compared with excluding the RIS and the GNN was 349%. This performance observation demonstrated the critical role of LiDAR-assisted RIS communications in enhancing the GNN’s ability to optimize beamforming.

### 4.3. Experiment 3: Varying User Pilot Sequence Lengths

The third experiment evaluated the impact of varying the user pilot sequence lengths on the achievable sumrate. The pilot lengths were tested across the set {L}={1,2,3,4,5,10,12,15,20}, with the downlink power fixed at 20 dBm and the number of RIS elements was set to N=100. The GNN framework was trained to map the pilot sequences and LiDAR-detected user locations to optimal beamforming and reflection coefficients. The evaluation was conducted to analyze the effects of varying the pilot length sequences on the LiDAR-GNN locations, when using a location-exclusive GNN, when using the LMMSE estimator, and when excluding the RIS entirely.

**Figure 8:**
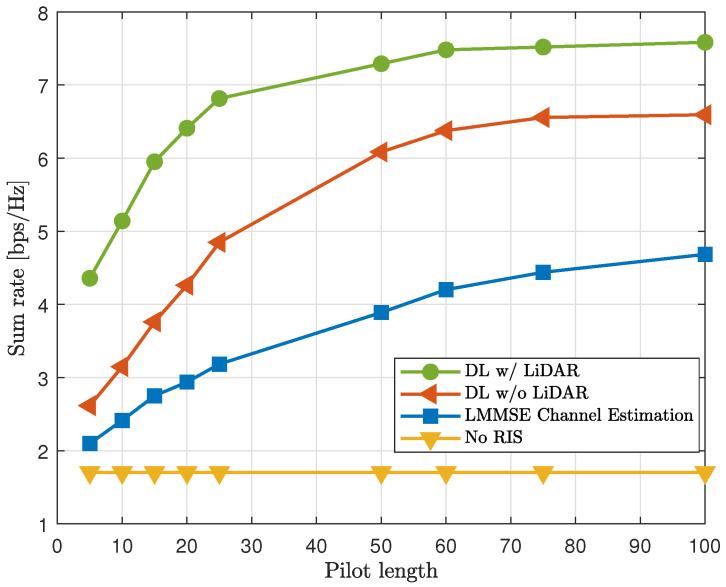
Sumrate comparison with varying pilot lengths within {L}.

The results, depicted in Figure 8, demonstrate that the GNN with LiDAR integration maintained superior performance across all pilot lengths. Shorter pilot lengths showed the most significant gains, highlighting the efficiency of the GNN in scenarios with a limited pilot overhead. The average sumrate improvement compared with the location-exclusive GNN was 22%. The proposed method outperformed the LMMSE channel estimator by 98% and achieved a remarkable 239% improvement compared with excluding the RIS. These findings confirm that incorporating LiDAR-detected locations is particularly advantageous in scenarios with a limited pilot overhead, validating the effectiveness of LiDAR-based localization in optimizing resource-constrained networks.

### 4.4. Experiment 4: Varying Number of RIS Elements

The fourth experiment explored the relationship between the number of RIS elements and the achievable sumrate. The pilot length was set to L=15, $P_{\mathrm{uplink}}=5$ dBm, and $P_{\mathrm{downlink}}=20$ dBm, while the number of RIS elements varied across the set {N}={100,125,150,175,200,225,250,275,300,325,350,375,400,425,450,475,500}. The evaluation was conducted to analyze the effects of increasing RIS elements with the LiDAR-GNN, the location-exclusive GNN, and the LMMSE estimator.

**Figure 9:**
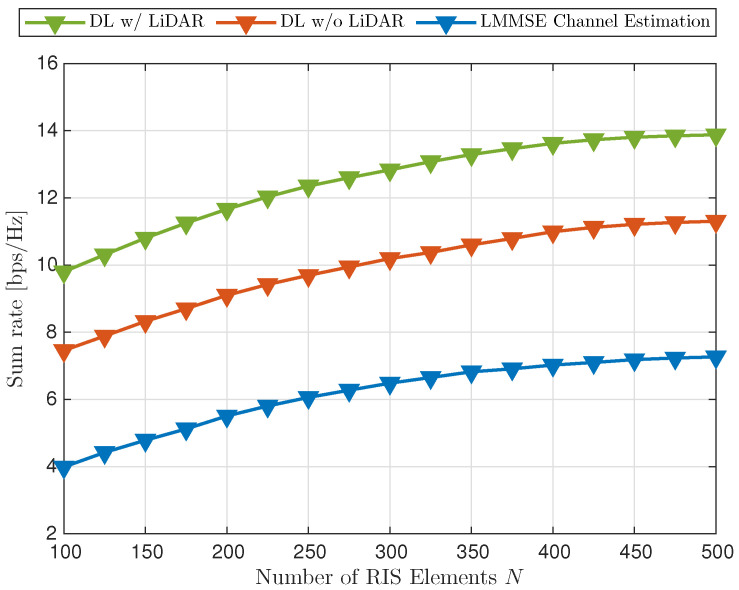
Sumrate comparison with varying RIS elements within {N}.

As shown in Figure 9, increasing the number of RIS elements significantly improved the performance. The performance gap between including and excluding the LiDAR data was 25.8%, and between the LiDAR-aided GNN and the LMMSE, the improvement was 101%. The sumrate improvement between excluding the LiDAR in the GNN and the LMMSE was 59.8%. This finding suggests that adding RIS elements consistently enhances the sumrate, indicating the substantial benefits of incorporating more RIS elements for optimizing network performance.

The results demonstrate that the LiDAR-aided GNN framework scales effectively with increasing RIS elements, leveraging the enhanced spatial resolution to optimize beamforming and reflection coefficients. These findings emphasize the importance of optimizing RIS configurations for achieving peak performance without an unnecessary resource overhead.

## 5. Discussion

The experimental findings presented in the previous section and summarized in Table 2 collectively illustrate the robustness and adaptability of the proposed LiDAR-aided GNN framework. The GNN achieved significant performance gains by integrating precise spatial information, particularly under challenging conditions, such as low downlink power, limited pilot sequences, or high-density user scenarios. The results highlight the substantial performance improvements enabled by LiDAR-aided user localization in RIS-assisted networks, addressing key challenges, such as interference mitigation and optimized beamforming. These findings underscore the potential of the proposed approach to enhance the network performance, which will be further discussed in terms of its implications, advantages, and areas for improvement.

### 5.1. Key Findings and Implications

Including LiDAR-detected user locations in the GNN framework led to significant improvements in the sumrate compared with both the location-exclusive GNN and the LMMSE baseline. The most pronounced benefits were observed under challenging conditions, such as low downlink power or short pilot sequences. These findings demonstrate that precise spatial information provided by LiDAR sensors enhanced the ability of the GNN to optimize beamforming and RIS phase coefficients, which resulted in more efficient resource utilization and improved communication quality.

Increasing the number of RIS elements enhances the sum-rate performance, but practical hardware constraints, such as controller complexity, power consumption, and manufacturing feasibility, limit the scalability in real-world systems. Specifically, the increasing number of control pins required for managing phase shifts complicates the design and power efficiency of the RIS controller. These challenges suggest an upper bound on the number of RIS elements that can be effectively implemented. The proposed approach primarily explores the theoretical potential of RIS with insights into performance trends and trade-offs.

Furthermore, the GNN’s permutation-invariant properties effectively handle the unordered nature of LiDAR point cloud data, enabling robust mapping from user locations to optimal configurations. This characteristic is particularly valuable in dynamic vehicular scenarios where user positions and environmental conditions frequently change. By dynamically adapting beamforming vectors based on LiDAR input, the system minimizes the latency and maximizes the spectral efficiency. The proposed approach is well-suited for real-world applications, such as urban vehicular networks and high-density user environments.

### 5.2. Advantages of LiDAR Integration

LiDAR technology offers unique advantages over other sensing modalities, such as cameras or radar. LiDAR’s ability to emit light enables precise localization in outdoor environments under varying weather and lighting conditions, ensuring reliable performance at night or in low-visibility conditions. While extreme conditions, like heavy rain or dense fog, can affect its range, LiDAR remains a dependable tool for capturing high-resolution 3D spatial data in vehicular networks [7,8].

Unlike traditional methods, the proposed framework bypasses this requirement, often relying on explicit CSI estimation with a significant pilot overhead. By leveraging accurate user localization through LiDAR, the system reduces the need for extensive pilot sequences, lowering the computational and signaling costs. This efficiency is particularly evident in scenarios with shorter pilot lengths or high user mobility, where traditional methods need help maintaining performance.

Additionally, the results indicate the scalability of the method. Even with increased pilot lengths or higher downlink power levels, the LiDAR-aided GNN maintained consistent performance advantages, suggesting that the framework can adapt to larger network scales or more complex scenarios.

### 5.3. Computational Complexity and Trade-Offs

The proposed method demonstrated significant performance improvements by leveraging LiDAR-based localization and GNN-based optimization for RIS-assisted networks. However, these advancements introduce practical computational challenges that must be addressed for real-time applications and scalability to larger networks.

Processing LiDAR data is resource-intensive, especially when using high-density point clouds to extract detailed spatial information. Frameworks such as SFA3D, which employ voxelized or BEV representations, require substantial computational resources for feature extraction and coordinate estimation. In the current setup, extracting user locations from LiDAR data takes an average of 50 ms per frame on an NVIDIA A40 8Q GPU. While this is adequate for many real-time applications, achieving sub-50 ms latency in scenarios with higher mobility or less capable hardware remains challenging. Optimization of the LiDAR-processing pipeline, such as through downsampling or approximate detection frameworks, could mitigate this bottleneck.

The GNN framework for beamforming and RIS phase coefficient optimization adds another layer of computational overhead. Training the GNN, which involves iterative backpropagation over multiple graph layers, required approximately 3.5 h until convergence (100 epochs) on an NVIDIA A40 8Q GPU in the experimental setup. This observation reflects the computational burden of handling RIS configurations and user spatial data. Inference, while faster, also introduces latency, with an average of 70 ms per scenario needed to generate beamforming vectors. These times are manageable for periodic updates in vehicular networks but may require optimization for faster response times in dynamic environments.

The training process presents challenges, including high memory requirements and careful tuning of hyperparameters, such as learning rates, graph layer depths, and the number of RIS elements. Improper configurations can lead to extended convergence times or suboptimal performance. In comparison, the LMMSE baseline, while computationally simpler, performs significantly worse in terms of the achievable sumrate and adaptability, highlighting the trade-off between computational efficiency and the benefits of spatial optimization provided by the GNN.

Several strategies can address these computational challenges. Leveraging advanced hardware, such as GPUs, TPUs, or FPGAs, can significantly reduce the training and inference times, primarily through techniques like batch inference for multi-user scenarios. Model optimization techniques, including pruning, quantization, and the development of lightweight graph architectures, can help lower resource requirements without compromising the performance. Additionally, pipeline optimizations, such as downsampling point clouds or using approximate detection frameworks for LiDAR data processing, can reduce latency. Finally, implementing asynchronous RIS updates based on prioritized user locations could help manage computational loads in high-mobility or high-density environments.

The proposed framework shows significant promise for enhancing RIS-assisted networks but requires careful consideration of these computational trade-offs in practical deployments. Optimizing the model and its execution pipeline will be essential to achieve the desired balance between performance and responsiveness in real-world applications. Future work will focus on refining the training and inference processes, exploring hardware-specific implementations, and evaluating multi-modal integrations further to enhance the scalability and efficiency of the framework.

### 5.4. Broader Implications

The transformative potential of LiDAR-aided GNN frameworks extends beyond RIS-assisted networks. By integrating LiDAR with other sensing modalities, such as cameras, radar, or inertial measurement units (IMUs), it is possible to develop multi-modal systems that leverage complementary strengths for enhanced user localization and environmental awareness. Future work could explore fusion algorithms to combine data from these modalities, improving the robustness and accuracy in diverse and dynamic environments. Additionally, integrating LiDAR data into edge computing architectures could reduce the latency and improve the scalability for real-time applications.

## 6. Future Work

The findings of this work open several promising avenues for future research to enhance the performance and applicability of LiDAR-aided GNN frameworks in RIS-assisted networks. Below, we outline key directions for improvement and exploration.

### 6.1. Optimization of Computational Efficiency

The proposed framework, while effective, introduces a computational overhead due to the processing of LiDAR data and the complexity of the GNN inference. Future research can focus on optimizing computational efficiency by the following:

- **Model pruning and quantization:** reducing the size and complexity of the GNN through techniques such as pruning redundant parameters and quantizing weights.
- **Lightweight neural architectures:** designing simplified GNN models tailored for RIS optimization without compromising performance.
- **Hardware acceleration:** exploring the use of GPUs, TPUs, or FPGAs to speed up LiDAR processing and GNN inference, enabling real-time deployment in resource-constrained environments.

### 6.2. Adaptive Beamforming and Phase Shift Optimization

The current approach assumes static GNN parameters and static users, which may limit its adaptability in highly dynamic environments. To address the adaptability challenge, future work can focus on the following:

- **Reinforcement learning:** integrating reinforcement learning techniques to enable adaptive beamforming and phase shift updates based on real-time feedback from the environment.
- **Online learning:** developing online learning algorithms that update the GNN model incrementally as new LiDAR data and communication metrics become available.
- **Mobility-aware optimization:** enhancing the framework to account for rapid user mobility, such as in vehicular or drone-assisted networks, ensuring consistent performance across dynamic scenarios.

### 6.3. Integration of Multi-Modal Sensing

LiDAR is a powerful tool for user localization, but its effectiveness can be further augmented by integrating additional sensing modalities. Future efforts could explore the following:

- **Fusion with other sensors:** Combining LiDAR data with cameras or radar creates a multi-modal sensing framework. This integration can provide complementary perspectives and improve the localization accuracy in complex environments.
- **Data fusion algorithms:** developing algorithms to effectively combine data from multiple sensors while managing uncertainties and measurement conflicts.
- **Robustness in diverse scenarios:** testing the framework in varied environments, such as urban areas, rural landscapes, and indoor settings, to evaluate its adaptability across different contexts.

## 7. Conclusions

This paper presents a novel LiDAR-aided GNN framework for optimizing RIS-assisted networks, leveraging high-resolution spatial data from LiDAR sensors to enhance the network performance. By incorporating LiDAR-detected user locations, the proposed approach achieved significant improvements in the sumrate compared with location-exclusive GNNs and baseline channel estimation methods, such as the LMMSE. The results demonstrated the framework’s effectiveness in optimizing beamforming vectors and RIS phase coefficients, particularly in challenging scenarios with a low downlink power, limited pilot sequences, or a high user mobility.

The GNN’s ability to process unordered LiDAR point cloud data using its permutation-invariant properties ensured robust and adaptive performance in dynamic and dense user environments. This capability highlights the framework’s potential for deployment in urban vehicular networks and other high-density scenarios. However, there are challenges—such as computational requirements associated with processing high-density LiDAR data and training graph-based models—for real-time implementation, particularly in large-scale or resource-constrained deployments.

Future work will address these computational challenges by exploring model-optimization techniques, such as pruning, quantization, and lightweight neural architectures. Additionally, hardware acceleration using GPUs; TPUs; or specialized hardware, such as FPGAs, will be investigated to reduce the latency during training and inference. Validation in real-world environments, including multi-modal integrations with sensors, such as radar or cameras, will also be a priority to assess the framework’s robustness and scalability under diverse conditions.

The proposed LiDAR-aided GNN framework offers a robust, scalable, and adaptable solution for improving the RIS-assisted network performance across various challenging scenarios. By addressing the computational trade-offs and validating its applicability in real-world deployments, this framework has the potential to advance the capabilities of next-generation wireless communication systems significantly.

## Figures and Tables

**Table 1:** Training parameters.

Parameter	Value
BS position	(100,100,0)
GNN training epochs	500
LiDAR-RIS vehicle position	(0,0,0)
Noise power ($P_{\mathrm{Noise}}$)	−100 dB
Number of BS antennas (*M*)	8
Number of samples	1000
Number of users (*U*)	5
Rician factor (ε)	10
Static downlink power ($P_{\mathrm{downlink}}$)	20 dBm
Static number of RIS elements (*N*)	100
Static pilot length (*L*)	15
Varying pilot length ({L})	{1,2,3,4,5,10,12,15,20}
Static uplink power ($P_{\mathrm{uplink}}$)	5 dBm
Varying downlink power ($\left\{P_{\mathrm{downlink}}\right\}$)	{0,2.5,5,7.5,10,12.5,15,17.5,20} dBm
Varying number of RIS elements ({N})	{100,125,150,175,200,225,250,275
	300,325,350,375,400,425,450,475,500}
Varying uplink power ($\left\{P_{\mathrm{uplink}}\right\}$)	{0,2.5,5,7.5,10,12.5,15,17.5,20} dBm

**Table 2:** Summary of results: performance gains across experiments.

Experiment	LiDAR-GNN vs. Location-Exclusive GNN (%)	LiDAR-GNN vs. LMMSE (%)	LiDAR-GNN vs. No RIS (%)
Varying $\left\{P_{\mathrm{downlink}}\right\}$	16.0	85.0	190.0
Varying $\left\{P_{\mathrm{uplink}}\right\}$	25.4	73.1	349.0
Varying {L}	22.0	98.0	239.0
Varying {N}	25.8	101.0	-

## Data Availability

The original contributions presented in this study are included in the article. Further inquiries can be directed to the corresponding author.

## Abbreviations

The following abbreviations are used in this manuscript:

BEV	Bird’s-Eye View
BS	Base station
CSI	Channel state information
FPN	Feature Pyramid Network
GNN	Graph Neural Network
IoT	Internet of Things
KFPN	Keypoint Feature Pyramid Network
LiDAR	Light Detecting and Ranging
LMMSE	Linear Minimum Mean Squared Error
LoS	Line of Sight
MIMO	Multiple-Input–Multiple-Output
MISO	Multiple-Input–Single-Output
MMSE	Minimum mean-squared error
NLoS	Non-Line of Sight
QoS	Quality of service
RIS	Reconfigurable Intelligent Surface
SFA3D	Super Fast and Accurate 3D Object Detection Framework
UAV	Unmanned aerial vehicle
UE	User Equipment
WPCN	Wireless-Powered Communication Network
